# Research of imaging in left Atrium: A Bibliometric Analysis

**DOI:** 10.2174/0115734056356151250604021748

**Published:** 2025-06-10

**Authors:** Can Cui, Jiang-Hua Zhu, Ya-Hong Tao, Zhen-Yi Zhao, Yun Peng, Minjing Zuo

**Affiliations:** 1 Department of Radiology, The Second Affiliated Hospital, Jiangxi Medical College, Nanchang University, Nanchang 330006, China; 2 Intelligent Medical Imaging of Jiangxi Key Laboratory, Nanchang 330006, China

**Keywords:** Left atrium imaging, Bibliometric analysis, CiteSpace, VOSviewer, Citation analysis, Atrial fibrillation, Cardiac Computed Tomography, Magnetic Resonance Imaging

## Abstract

**Background:**

The evaluation of the left atrial (LA) by imaging is becoming increasingly essential due to its significant role in numerous diseases. This study aimed to analyze and summarize research on LA imaging in the past 20 years through bibliometric analysis and offer insights into future research prospects.

**Methods:**

The Web of Science (WOS) core collection database was retrieved for literature in LA imaging research from 2004 to 2023. Subsequently, the literature was processed and visualized by the VOSviewer and CiteSpace. VOSviewer was used to create cooperation networks for countries/regions and institutions. CiteSpace was used to analyze burst keywords in citation analysis.

**Results:**

A total of 3664 articles published in this field between January 2004 and December 2023 were analyzed. The number of published articles is increasing year by year. The USA contributed the most articles (1072). Hugh Calkins (44) was the most productive author with the highest publications.

**Conclusion:**

Over the past 20 years, research on LA imaging has grown rapidly. The results of the present study provide insights into the field’s status and indicate the research hotspots. In recent years, research on left atrial appendage occlusion (LAAO) and LA strain has been notably focused, which is expected to remain a prominent topic in future research.

## 1 INTRODUCTION

The LA serves as a crucial link between the left ventricle and the pulmonary veins [1] and plays a vital role in the process of filling the left ventricle and in maintaining overall cardiac function. The evaluation of the LA is essential, particularly in patients with atrial fibrillation (AF) [2]Assessing the structure and function can help stratify risk, guide treatment, and predict prognosis [3]. Clinical evaluation of LA includes biochemical markers like NT-proBNP, ANP, BNP, *etc* [4, 5]. The alterations of these biochemical markers involve a variety of molecular and pathological mechanisms [6]. Imaging assessment is also an important method of LA evaluation [7], such as ultrasound, Cardiac Computed Tomography (CT), and Cardiac Magnetic Resonance Imaging (MRI). Imaging can offer a thorough evaluation of the LA structure and function [8], thus offering valuable information for the treatment and prognosis. For example, CT can clearly show the structure and anatomical variation of the pulmonary veins [9, 10], which is of great significance for the preoperative evaluation of radiofrequency ablation of AF.

Bibliometrics is a method of statistical analysis [11] that examines the authors, journals, citation frequency, and publication year of literature [12]. It is used to discover patterns within the literature, summarize research hotspots, and identify development trends in the analyzed disciplines. There are many studies on LA, but quantitative analysis is still lacking. This study aimed to analyze and visualize the research on LA imaging in the past 20 years through bibliometric analysis, provide insights into the field’s status, and indicate the research hotspots.

## 2 METHODS

We conducted a literature search on the WOS Core Collection on 17 July 2024 using the following search formula: (TS= ((“Left Atrium” or “Left atrial”) AND (“CT” or “MRI” or “Magnetic Resonance Imaging” or “PET” or “positron emis-sion tomography” or “computed tomography” or “Coronary angiography” or “radiography” or “ultrasonography”)) AND (FPY=2004-2023). The criteria for literature inclusion were as follows: (I) LA imaging as the topic of study, (II) articles and reviews, and (III) English-language literature. Meanwhile, the exclusion criteria were as follows: (I) meeting abstract, editorial material, proceeding paper, and letter; (II) literature not published between January 2004 and December 2023, and (III)the research field of the literature does not match the topic, such as veterinary sciences, agriculture, and zoology. The process is shown in Fig. (**1**).

## 2.1 Data Analysis

CiteSpace (version 6.2) and VOSviewer (version 1.6.2) were applied for bibliometric and visualization analysis of the included literature. VOSviewer was used to create cooperation networks for countries/regions and institutions. Citespace presents the research structure and development trend of a certain discipline in specific fields [13]. which was used to analyze citation burst keywords.

## 3 RESULTS

### 3.1 Temporal Distribution of the Literature

Fig. (**2**) is a graph of the number of publications drawn by Excel, which depicts the trend of the number of publications per year. The number of published articles has been increasing year by year from 2004 to 2023 and reached 370 in 2023.

### 3.2 Contribution of Countries and Regions

Table **1** shows the top 10 countries with the largest number of publications. The United States, China, and Germany ranked in the top three, with 1072, 445, and 416 articles, respectively. Fig. (**3**) shows the cooperation map of countries or regions in LA imaging studies. The United States, the United Kingdom, South Korea, Australia, and Lithuania cooperate closely; China has more cooperation with Germany, the United States, the United Kingdom, Australia, and France.

### 3.3 Distribution of Research Institutions

Fig. (**4**) shows the institution’s cooperation network diagram by using VOSviewer. It demonstrated that the cooperation between different institutions within China was intense, among which Shanghai Jiao Tong University established close cooperation with Wuhan University and capital Medical University. However, there is less cooperation with institutions abroad.

### 3.4 Author Analysis

Table **2** lists the top 10 authors with the highest number of publications in the field of LA imaging. The author with the highest number of publications was Hugh Calkins, with a total of 44 publications, followed by Nassir F. Marrouche and Eugene Kholmovski with 39 and 36 publications. We also conducted an h-index analysis of the authors, and the results showed that Yun-Yu Chen had the highest h-index (18), followed by Hugh Calkins.

### 3.5 Journal Distribution

As shown in Table **3**, the Journal of Cardiovascular Electrophysiology is the most productive in the field of LA imaging, with a total of 171 articles. It was followed by Europace (124), the International Journal of Cardiovascular Imaging (110), Heart Rhythm (103), and the International Journal of Cardiology (95).

### 3.6 Analysis of Keyword Burst

As shown in Fig. (**5**), a pulmonary vein was the strongest burst word with an intensity of 22.92, followed by radiofrequency ablation with an intensity of 20.03. In 2003-2017, the strongest citation bursts included: pulmonary vein, radiofrequency ablation, initiation, anatomy, complication, *etc*. Keywords that appeared after 2017 and continued to have the strongest citation bursts include occlusion, LAAO, strain, and clinical outcome.

## 4 DISCUSSION

The study analyzed the use of imaging in LA through bibliometrics. The number of published articles in this field has increased steadily from 35 in 2004 to 370 in 2023. This upward trend suggests that LA imaging research is garnering significant attention among scholars.

The top three countries with the largest number of publications are the United States, China, and Germany, indicating their strong interest in research in this field. In institutional analysis, Harvard University ranks first. Seven of the top 10 institutions are from the United States, which also shows that the United States is leading in this field. The United States has very close exchanges with other countries, which have also contributed to the development of the field of LA imaging. Analysis of cited journals reveals that Circulation is the most frequently cited journal in the field of LA imaging research, underscoring its popularity and authoritative standing.

**Fig. (5):**
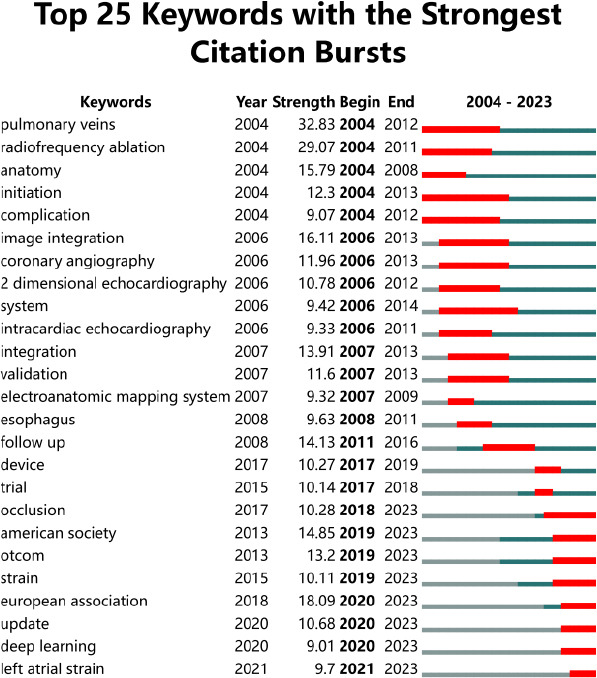
Top 25 keywords with the strongest citation bursts.

Keyword burst analysis can reflect the main research hotspots and their trends in a specific period. According to (Fig. **5**), the keyword burst is divided into two phases. The first phase is 2004-2017, emphasis is placed on the anatomy of the LA and pulmonary veins, and some preliminary imaging techniques. AF is a highly prevalent cardiovascular disease that is closely related to LA remodeling [14]. Hugh Calkins and his team demonstrated that radiofrequency ablation for the treatment of AF reports higher efficacy rates than anti-arrhythmic drug therapy and a lower rate of complications [15]. Radiofrequency ablation requires only limited fluoroscopy, is catheter-guided, and is achieved using an electroanatomic mapping system [16]. It is important to note that during radiofrequency ablation, the relative position of the esophagus to the posterior wall of the LA needs to be identified to avoid complications such as esophageal injury during surgery [17]. Michel Haissaguerre *et al*. have shown that the spontaneous initiation of AF by ectopic beats originating in the pulmonary veins [18]. These ectopic rhythms originate from the muscle bundles that extend from the atria to the pulmonary veins, so pulmonary vein isolation is the cornerstone of radiofrequency ablation. Besides, the changes in the number and location of pulmonary veins correlated with the outcome of AF radiofrequency catheter ablation procedures. Recommended by Kirchhof *et al*. in the 2016 ESC Guidelines [19] for the management of AF, developed in collaboration with EACTS, routine LA size and anatomy assessment in patients with AF is essential. LA size is usually assessed using standard 2D echocardiography [20] in the past. With the development of imaging technology, intracardiac echocardiography [21]. It is complementary to transesophageal echocardiography and is much better tolerated by patients, helping to meet the growing need for monitoring of patient anatomy, catheter placement, and intraoperative complications such as pericardial effusion or thrombosis.

In the last few years, it has become possible to integrate different imaging modalities to guide catheter ablation procedures for AF [22]. There are now a variety of specialized hybrid imaging systems or software image co-registration techniques [23] that combine anatomical information from fluoroscopy, CT, MRI, or intracardiac echocardiography with information provided by electroanatomic mapping to guide catheter ablation of AF [24]. The development of imaging methods and techniques has led to a better understanding of the LA and pulmonary veins [25].

From 2017 to 2023, the focus shifted towards clinical efficacy and patient prognosis, as well as devices for LAAO. Furthermore, the LA strain emerged as a significant research topic [26]. Left atrial appendage thrombosis stands as the primary cause of thromboembolic stroke in almost 90% of AF patients [27], LAAO represents a swiftly evolving technique for individuals unable to sustain long-term oral anticoagulation [28]. LAAO emerged in 1949 as a surgical procedure, with percutaneous closure first conducted on human patients in 2001. There are many LAAO devices, such as Watchman, PLAATO, LARIAT, WaveCrest, and Amplatzer [29]. Among them, Watchman is the best-studied LAAO device. In 2017, a clinical trial called PREVAIL and PROTECT AF Trials showed that LAAO with Watchman devices prevents stroke in patients with nonvalvular AF to a similar extent to oral anticoagulation with warfarin [30]. In addition, LAAO causes less disability or death than warfarin [31]. Recent studies from American scholars have shown that LAAO surgery has significant advantages over oral anticoagulant therapy in improving long-term cognitive function, regardless of gender and type of AF. Imaging modalities like transesophageal echocardiography (TEE) or LA CT serve as the primary imaging techniques for post-LAAO assessment [32]. Typically conducted 1 to 6 months post-surgery. These modalities can detect peri-device leakage, device-related thrombosis, and device embolism to inform subsequent management decisions.

LA strain has garnered attention as a significant area of research in recent years [33]. Strain denotes the myocardial deformation during the cardiac cycle, representing the percentage change in myocardial length from diastolic relaxation to systolic contraction [34]. Echocardiographic dot tracking technology, LA CT, and cardiac MRI all enable the evaluation of LA strain, providing a comprehensive assessment of LA storage, conduit, and contractile pump functions. The strain rate refers to [35] the speed of myocardial deformation, which can more accurately and sensitively reflect the early functional impairment of the myocardium. There is growing interest in the use of artificial intelligence (AI) in LA imaging, especially deep learning-based AI. Deep learning was more reproducible than imaging measurements [36]. Deep learning can also integrate latent information from imaging that may inform disease risk. DROID, a deep learning echocardiographic interpretation model built by Emily Lau and her team [37], accurately quantifies standard measurements of LA structure and function [38] and can combine information from other heart chambers to generate predictions of LA size, which may provide additional predictive value for disease risk. Medical imaging provides information in a noninvasive, reproducible manner, has grown significantly in importance in clinical decision-making [39].

This bibliometrics article has certain limitations. Firstly, citation data can be influenced by factors such as language preferences and subject selections, potentially impacting the reliability of bibliometric analysis. Secondly, the data were retrieved solely from the WOS, excluding articles from other databases, potentially resulting in the omission of relevant literature. Lastly, the collection and statistical analysis of literature data may vary among individuals and could be subjectively influenced by personal perceptions.

## CONCLUSION

The top 3 countries with the largest number of publications are the United States, the United Kingdom, and Germany, which suggests that these countries are interested in this area and have a lot of research results. In recent years, research on LAAO and LA strain has been notably focused and garnered considerable attention. The ongoing trend of utilizing multimodal LA imaging to evaluate the prognosis and LA strain post-LAAO is expected to remain a prominent topic in future research.

## Figures and Tables

**Fig. (1):**
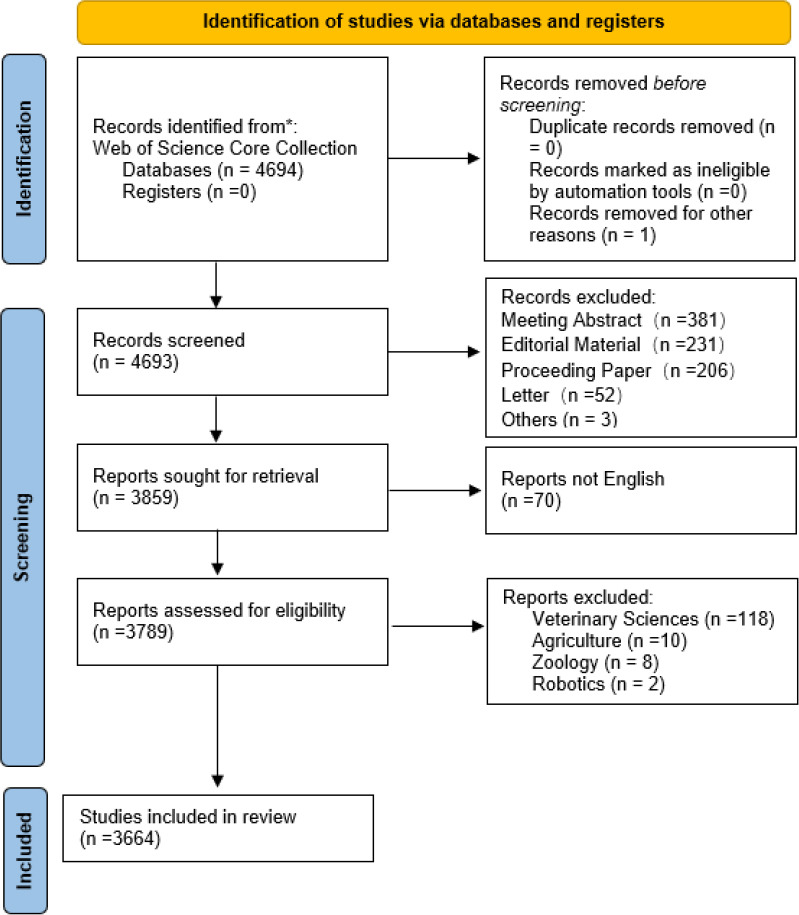
Literature screening flow chart.

**Fig. (2):**
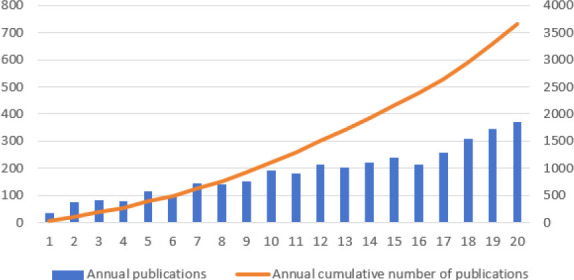
The annual number of relevant publications and the annual cumulative number of articles published from 2004 to 2023.

**Fig. (3):**
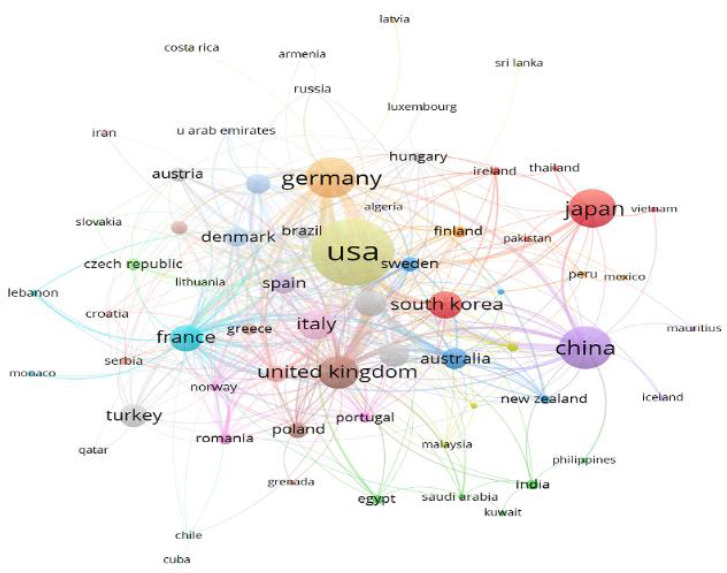
Cooperation network of countries/regions in LA imaging studies. The nodes with different colors represent different countries and regions, and the size of the nodes represents the number of publications. Lines represent the connections between the various nodes, and the more lines there are, the more cooperation between the two countries or regions.

**Fig. (4):**
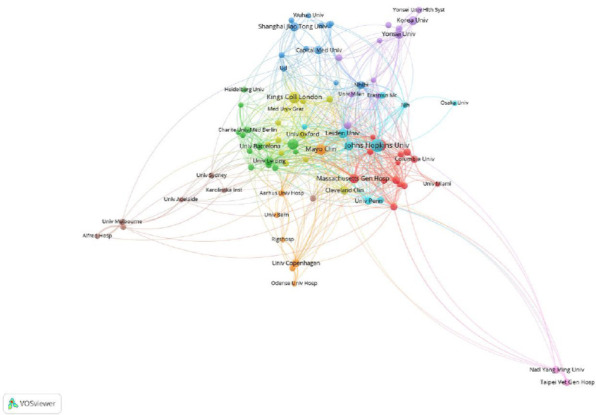
Cooperation network of institutions. The circle size represents the publication volume, while the line thickness represents a research connection between two institutions based on their co-authorship status. The various colors represent different cooperation clusters, and institutions grouped within the same cluster may possess greater potential for collaborative relationships.

**Table 1:** Top 10 countries by the number of publications.

Rank	Countries	Count
1	USA	1072
2	China	445
3	Germany	416
4	Japan	370
5	UK	271
6	Italy	233
7	South Korea	191
8	France	166
9	Netherlands	161
10	Canada	150

**Table 2:** The top 10 authors with the highest number of publications.

Rank	Authors	Count	Country/Region			H-index
1	Hugh Calkins	44	USA			127
2	NassirF. Marrouche	39	USA			57
3	Eugene Kholmovski	36	USA			33
4	Yun-Yu Chen	35	Chinese Taipei			129
5	Hui-Nam Pak	35	South Korea			47
6	Saman Nazarian	34	USA			55
7	Gerhard Hindricks	30	Germany			84
8	Boyoung Joung	28	South Korea			47
9	Joao A. C. Lima	28	USA			83
10	Ronald D Berger	25	USA			64

**Table 3:** Top 10 most productive journals.

Rank	Journals	Count	IF2023
1	Journal of Cardiovascular Electrophysiology	171	2.7
2	Europace	124	6.1
3	International Journal of Cardiovascular Imaging	110	2.1
4	Heart Rhythm	103	5.5
5	International Journal of Cardiology	95	3.5
6	Journal of Interventional Cardiovascular Electrophysiology	89	1.8
7	Frontiers In Cardiovascular Medicine	79	3.6
8	European Heart Journal Cardiovascular Imaging	65	6.2
9	Pace Pacing and Clinical Electrophysiology	62	1.7
10	Echocardiography A Journal of Cardiovascular Ultrasound and Allied Techniques	61	1.5

## Data Availability

The data and supportive information are available within the article.

## LIST OF ABBREVIATIONS

LA: Left Atrial
WOS: Web of Science
LAAO: Left Atrial Appendage Occlusion
AF: Atrial Fibrillation
CT: Computed Tomography
MRI: Magnetic Resonance Imaging

## STANDARDS OF REPORTING

PRISMA guidelines and methodology were followed.

## SUPPLEMENTARY MATERIALS

PRISMA checklist is available as supplementary material on the publisher’s website along with the published article.
